# Truncated C-terminus of fibrillin-1 induces Marfanoid-progeroid-lipodystrophy (MPL) syndrome in rabbit

**DOI:** 10.1242/dmm.031542

**Published:** 2018-04-09

**Authors:** Mao Chen, Bing Yao, Qiangbing Yang, Jichao Deng, Yuning Song, Tingting Sui, Lina Zhou, HaoBing Yao, Yuanyuan Xu, Hongsheng Ouyang, Daxin Pang, Zhanjun Li, Liangxue Lai

**Affiliations:** 1 Jilin Provincial Key Laboratory of Animal Embryo Engineering, Jilin University, Changchun 130062, China; 2 Key Laboratory of Regenerative Biology, Chinese Academy of Sciences, and Guangdong Provincial Key Laboratory of Stem Cells and Regenerative Medicine, South China Institute for Stem Cell Biology and Regenerative Medicine, Guangzhou Institutes of Biomedicine and Health, Chinese Academy of Sciences, Guangzhou, Guangdong 510530, China

**Keywords:** Fibrillin-1, CRISPR/Cas9, Marfanoid-progeroid-lipodystrophy (MPL) syndrome, Rabbit

## Abstract

Various clinical differences have been observed between patients with the *FBN1* gene mutation and those with the classical Marfan phenotype. Although *FBN1* knockout (KO) or dominant-negative mutant mice are widely used as an animal model for Marfan syndrome (MFS), these mice cannot recapitulate the genotype/phenotype relationship of Marfanoid-progeroid-lipodystrophy (MPL) syndrome, which is caused by a mutation in the C-terminus of fibrillin-1, the penultimate exon of the *FBN1* gene. Here, we describe the generation of a rabbit MPL model with C-terminal truncation of fibrillin-1 using a CRISPR/Cas9 system. *FBN1* heterozygous (*FBN1* Het) rabbits faithfully recapitulated the phenotypes of MFS, including muscle wasting and impaired connective tissue, ocular syndrome and aortic dilation. Moreover, skin symptoms, lipodystrophy, growth retardation and dysglycemia were also seen in these *FBN1* Het rabbits, and have not been reported in other animal models. In conclusion, this novel rabbit model mimics the histopathological changes and functional defects of MPL syndrome, and could become a valuable model for studies of pathogenesis and drug screening for MPL syndrome.

## Abstract (precis)

**Summary:** A novel genetically engineered rabbit model of MPL syndrome, generated by CRISPR/Cas9-mediated mutation of *FBN1*, mimics the histopathological changes and functional defects of MPL syndrome seen in the clinic.

## INTRODUCTION

Different mutations in the *FBN1* gene lead to a wide range of diseases, such as Marfan syndrome (MFS), Weill–Marchesani syndrome, acromelic dysplasias, stiff skin syndrome and Marfanoid-progeroid-lipodystrophy (MPL) syndrome (Pepe et al., 2016). The *FBN1* gene (Gene ID: 100350931) encodes fibrillin-1, a major component of 10-12 nm diameter microfibrils. These microfibrils are found in the periphery of elastic fibers and elastin-free bundles, including the ciliary zonule, the elastic fibers in connective tissue (Neptune et al., 2003; Sakai et al., 2016). The *FBN1* gene consists of 66 exons and has seven main protein domains, including a calcium-binding domain (cbEGF), noncalcium-binding domain, TGF-β-binding protein-like domain, hybrid domain, proline-rich domain, N-terminal domain and C-terminal domain (Davis and Summers, 2012).

MPL syndrome is a novel fibrillinopathy and a disease that has very rarely been reported in the clinic. All MPL patients carry mutations in exon 64, which leads to a premature stop codon in the C-terminus of *FBN1* (Garg and Xing, 2014; Goldblatt et al., 2011; Graul-Neumann et al., 2010; Jacquinet et al., 2014; Romere et al., 2016; Song et al., 2012). Thoracic aortic aneurysm/dissection (TAAD), ectopia lentis, and systemic features with score ≥7 are the main clinical criteria of classical MFS, while the most apparent features of MPL syndrome are a progeroid appearance and lipodystrophy (Chandra et al., 2015; Jacquinet et al., 2014; Passarge et al., 2016). Of note, the C-terminus of fibrillin-1 contains a highly conserved propeptide that undergoes furin-mediated processing (Lönnqvist et al., 1998). Furthermore, the C-terminal propeptide is required for fibrillin-1 secretion and blocks premature assembly through linkage to the domains of cbEGF41-43 (Jensen et al., 2014). In addition, previous studies report that multimerization of the fibrillin-1 C-terminus into bead-like structures enables self-assembly (Hubmacher et al., 2008). Although the C-terminus was required for secretion and self-assembly of fibrillin-1 at the cellular level (Hubmacher et al., 2008; Jensen et al., 2014), the pathogenesis of MPL syndrome is not clear in the animal model.

At present, multiple animal models with mutations in the *FBN1* gene exist, including models in mice (Carta et al., 2006; Granata et al., 2016; Judge et al., 2004; Pereira et al., 1997, 1999; Sengle et al., 2012; Singleton et al., 2005), pigs (Umeyama et al., 2016) and cattle (Besser et al., 1990; Singleton et al., 2005). Most of the studies on pathogenesis and treatment have been carried out in *FBN1* mutant mice. For example, dysregulation of TGF-β activation was observed and losartan was applied for prevention of aortic aneurysm in a mouse model (Habashi et al., 2006; Neptune et al., 2003). The rabbit is a classic animal model species for cardiovascular diseases such as atherosclerosis (Getz and Reardon, 2012). In addition, owing to their large eyes, rabbits are a commonly used animal model for eye disease research. Thus, the rabbit could be used as a suitable animal model for MPL syndrome, displaying the typical pathological characteristics of aorta and eye observed in MPL patients.

In this study, we describe a novel rabbit model with a truncated C-terminus of fibrillin-1, which was achieved through cytoplasm microinjection of Cas9 mRNA and single guide RNAs (sgRNAs) into rabbit zygotes. The heterozygous rabbit model recapitulates muscle wasting, ocular syndrome, aortic dilation and lipodystrophy of MPL syndrome in the clinic, and shows decreased assembly of microfibrils in the extracellular matrix. As such, it will be a valuable model for studies of pathogenesis and drug screening.

## RESULTS

### Generation and MPL phenotype of *FBN1* Het rabbits

**Fig. 1.**
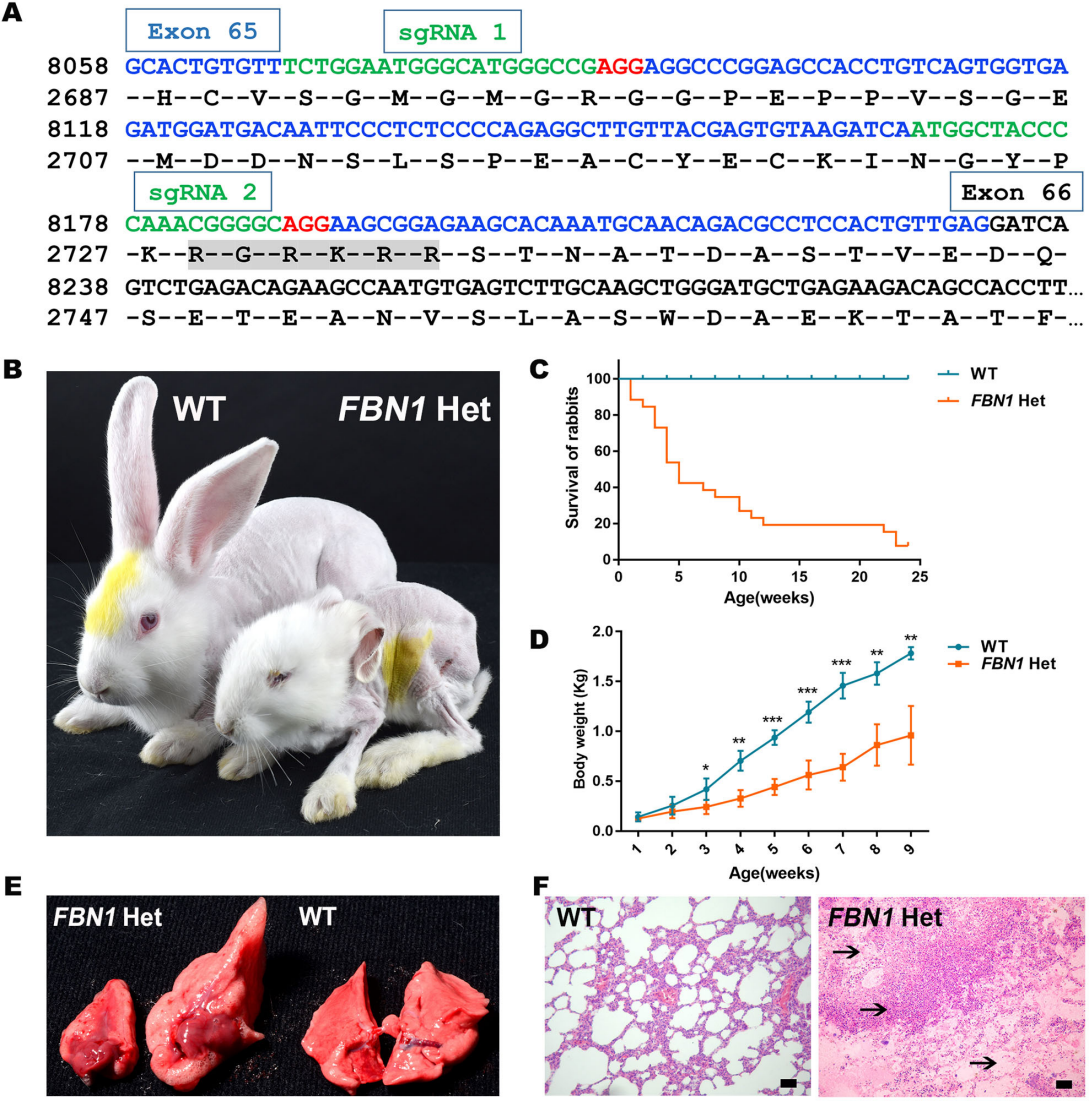
**Generation and MPL phenotype characterization of *FBN1* Het rabbits.** (A) Design of sgRNAs targeting the C-terminus of the rabbit *FBN1* gene. Blue bases represent exon 65 of *FBN1*; target sites of the two sgRNA sequences, sgRNA1 and sgRNA2, are highlighted in green; protospacer-adjacent motif (PAM) sequence is highlighted in red; ‘RGRKRR’ with gray background is the furin enzyme recognition site. (B) Photograph showing the bent ears, thin face and eyelid abnormalities of a *FBN1* Het rabbit. (C) Survival curves show the high mortality of *FBN1* Het rabbits (WT, *n*=9; KO, *n*=26). (D) Weight comparison results showing growth retardation in the *FBN1* Het rabbits (*n*=6). (E) Photographs showing the swollen lungs of *FBN1* Het rabbits (left, F0-9) compared with those of WT rabbits (right). (F) H&E stain showing pulmonary infection and edema (arrows) in an *FBN1* Het rabbit (F0-9). Data are presented as mean±s.e.m. and analyzed using Student's *t*-tests with GraphPad Prism software 6.0. *P<0.05; **P<0.01; ***P<0.001. Scale bars: 50 μm.

The C-terminus of fibrillin-1, which contains a glucogenic hormone, correlates with lipodystrophy in MPL patients (Jacquinet et al., 2014; Romere et al., 2016). In order to disrupt the function of *FBN1*, two sgRNAs targeting the C-terminus of fibrillin-1 were designed (Fig. 1A). To generate the *FBN1* mutant rabbits, a total of 197 injected zygotes were transferred into the oviducts of four surrogate rabbits. All surrogates carried to term and gave birth to 28 live pups (Table S1). The mutation detection results showed that 26 of the 28 (93.0%) newborn pups carried the mutation (Fig. S1). An off-target assay showed that the CRISPR/Cas9 system did not induce undesirable off-target effects in the *FBN1* mutant rabbits (Fig. S2). These results demonstrated that mutations in *FBN1* can be achieved via the CRISPR/Cas9 system with high efficiency in rabbits.

Patients with MPL syndrome showed thinness of body, progeroid symptoms, respiratory alterations and early growth retardation in the clinic (Jacquinet et al., 2014). Therefore, to determine the effects of *FBN1* mutation and characterize the phenotypes of *FBN1* Het rabbits, body weight, mortality rate and histopathology were compared between the WT and *FBN1* Het rabbits. As shown in Fig. 1B, *FBN1* Het rabbits exhibited apparent progeroid facial features, including thin face, eyelid anomalies, slender limbs, enophthalmus and bent ears, characteristics identical to the progeroid symptoms seen in human MPL syndrome (Ting et al., 2010). Compared with the WT controls, lower body weight was also determined in the founder generation (F0) of *FBN1* Het rabbits (Fig. 1C) (*P<*0.05). As expected, a significantly high mortality rate was observed in *FBN1* Het rabbits (Fig. 1D), and pulmonary infections, pneumothorax and ascending aortic dilatation were found in the dead rabbits (Fig. 1E,F). Thus, it was speculated that the possible cause of death was aortic root dilatation and MPL lung phenotype. These observations suggest that truncation of *FBN1* C-terminus induces the typical phenotypes of MPL syndrome in *FBN1* Het rabbits.

### Hereditary of the *FBN1* Het rabbits

In order to obtain a sufficient number of rabbits for detailed phenotypic characterization of MPL syndrome, a sexually mature F0 founder (F0-7, male, genomic chimera) was mated with a WT female and the genotypes were determined by PCR and T-cloning (Fig. S3A). The results revealed that *FBN1* mutations were detected in all 18 F1 rabbit pups, and the frame-shift mutation was determined in 12 pups (Fig. S3B,C).

We further examined the heritability of MPL syndrome in the F1 *FBN1* Het rabbits (Table S2). The results showed that the phenotypes of MPL syndrome – including bent ears, thin face and eyelid anomalies (Fig. S3A), growth retardation and high mortality (Fig. S3D) – were seen in the F1 generation rabbits (*FBN1^+/−2bp^*), but not in the in-frame mutated rabbits (*FBN1^+/−3bp^*, *FBN1^+/−18bp^*). Thus, our data confirm that the phenotypes of MPL are dominantly inherited in the *FBN1* Het rabbits.

### Reduced expression and secretion of fibrillin-1 in *FBN1* Het rabbits

**Fig. 2.**
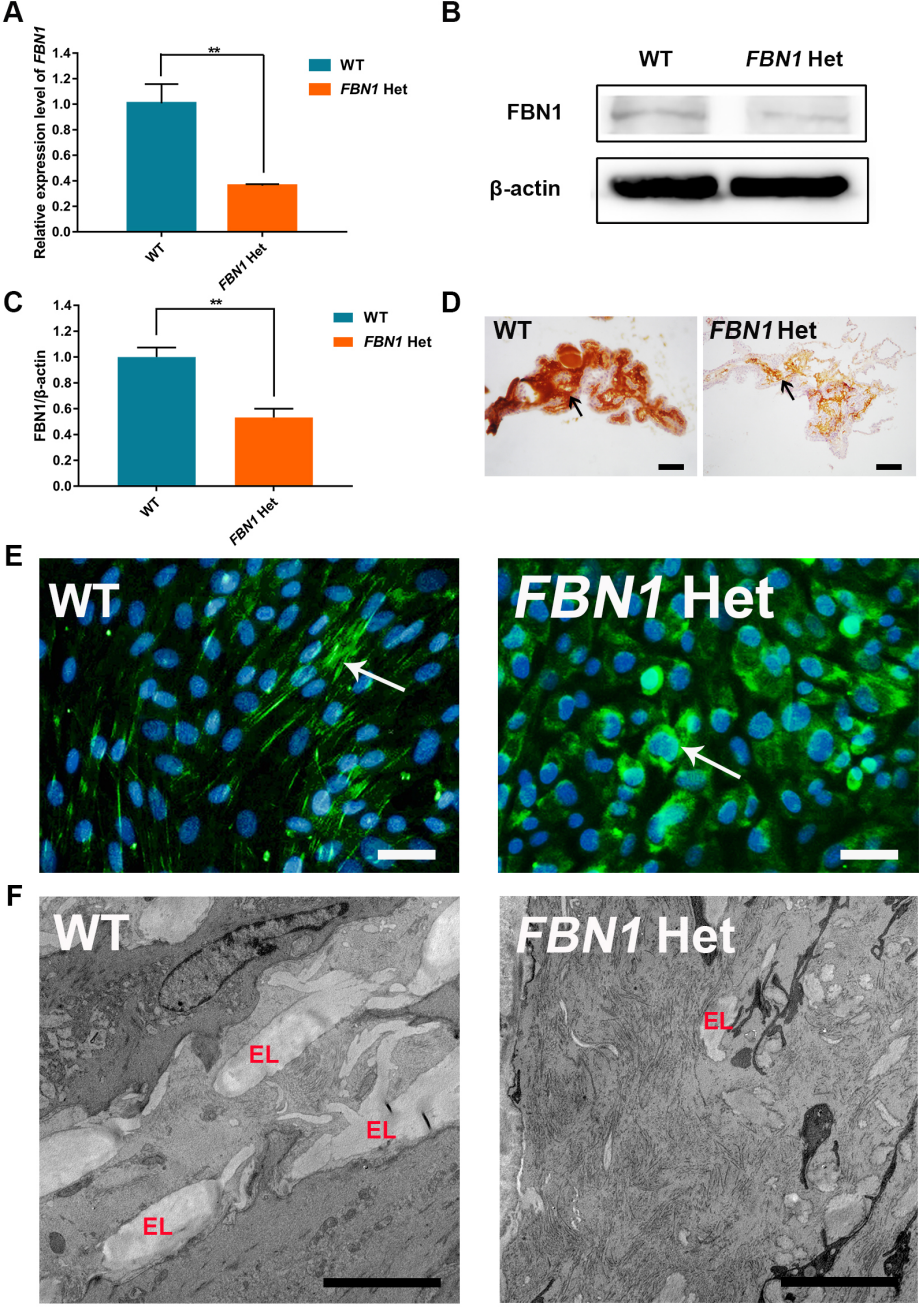
**Reduced expression and secretion of fibrillin-1 in *FBN1* Het rabbits.** (A) qPCR results show the significantly decreased expression of *FBN1* in *FBN1* Het rabbits. *n*=5. (B) Western blot showing significantly decreased expression of fibrillin-1 protein in *FBN1* Het rabbits. (C) Gray-scale analysis results show significantly decreased expression of fibrillin-1 protein in *FBN1* Het rabbits. *n*=5. (D) IHC showed decreased expression of fibrillin-1 (arrow) in the ciliary body of *FBN1* Het rabbits. (E) Immunofluorescence showed decreased secretion and assembly of microfibrils (arrow) into the extracellular matrix in *FBN1* Het rabbits. (F) Transmission electron microscopy showed reduced elastin (EL) in the thoracic aorta of *FBN1* Het rabbits. Data are presented as mean± s.e.m. and analyzed using Student's *t*-tests with GraphPad Prism software 6.0. **P<0.01. Scale bars: 50 μm.

The reduced expression of the *FBN1* allele with premature termination codons has been widely reported in the clinic (Hutchinson et al., 2003; Schrijver et al., 2002). In addition, quantitative mRNA analysis showed that nonsense-mediated decay (NMD) modulated the expression of truncated *FBN1* (Caputi et al., 2002; Magyar et al., 2009)*.* In fact, significantly reduced *FBN1* mRNA expression was observed in the *FBN1* Het rabbits compared with the WT rabbits (Fig. 2A) (*P*<0.01). These results were also confirmed by western blotting, gray-scale analysis and immunohistochemical analysis (IHC) of the ciliary body at the protein level (Fig. 2B-D) (*P*<0.01). The results suggested that reduced expression mediated by NMD contributes to generation of MPL syndrome in *FBN1* Het rabbits.

One report suggested that the loss of the C-terminal propeptide leads to reduced secretion of fibrillin-1 (Jensen et al., 2014). The assembly of microfibrils was determined by secretion of fibrillin-1. To determine the influence of truncated C-terminus on the assembly of microfibrils, dermal fibroblasts were isolated and cultured for 5 days. The results showed that fibrillin-1 was secreted and assembled into extracellular microfibrils in WT control fibroblasts, while intracellular retention occurred in fibroblasts of *FBN1* Het rabbits (Fig. 2E). In addition, the elastin deposited on the fibrillin microfibril scaffold was significantly reduced in the thoracic aorta of *FBN1* Het rabbits compared with that in WT rabbits, as observed by transmission electron microscopy (Fig. 2F) (Karimi and Milewicz, 2016).

### Eye symptoms of *FBN1* Het rabbits

**Fig. 3.**
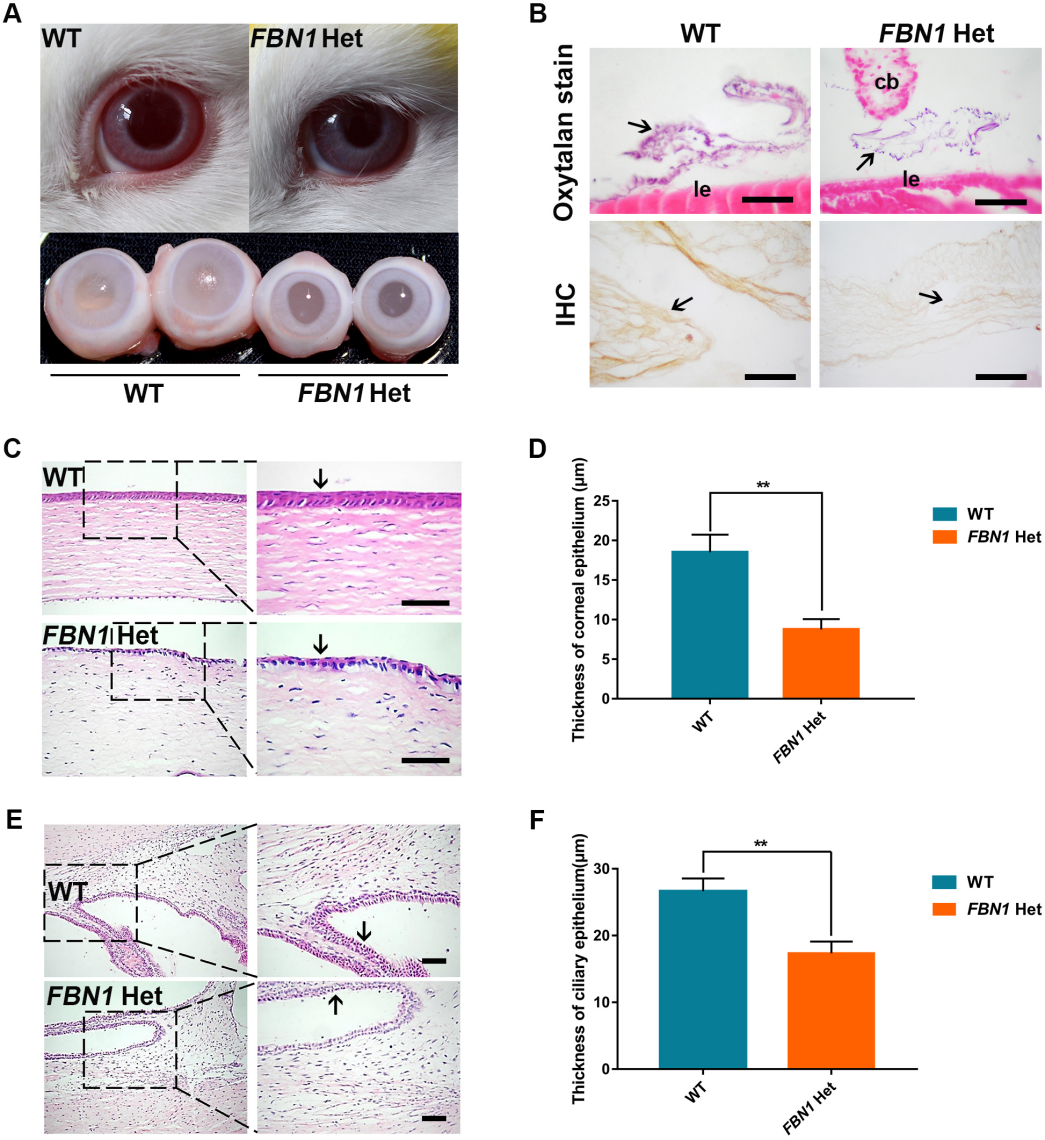
**Ocular symptoms in *FBN1* Het rabbits.** (A) The appearance and anatomy of *FBN1* Het rabbit eyes, showing the enophthalmus, eyelid abnormalities and microphthalmia. (B) Oxytalan staining and IHC show the presence of zonules (arrows) and decreased fibrillin-1 in zonules of *FBN1* Het rabbits. cb, ciliary body; le, lens epithelium. (C) H&E stain showing the defect in corneal epithelium (arrows) in *FBN1* Het rabbits. (D) Statistical comparison of the thickness of the corneal epithelium in *FBN1* Het rabbits and WT controls. (E) H&E stain showing the defect in nonpigmented epithelium in the ciliary body (arrow) in *FBN1* Het rabbits. (F) Statistical comparison of the thickness of nonpigmented epithelium in the ciliary body in *FBN1* Het rabbits and WT controls. Data are presented as mean±s.e.m. and analyzed using Student's *t*-tests with GraphPad Prism software 6.0. **P<0.01. *n*=6. Scale bars: 1 μm.

It is known that tissue microfibrils form in the ocular zonule, and fibrillins are the major constituent of tissue microfibrils. The most prominent ocular features of MPL syndrome patients are myopia, ectopia lentis, eyelid anomalies and enophthalmus (Liu et al., 2011; Loeys et al., 2010). To investigate the ocular zonule and ocular features of *FBN1* Het rabbits, the eyes were isolated from *FBN1* Het and WT rabbits and used for histopathological assay. We observed enophthalmus, eyelid anomalies and smaller eyeballs in the *FBN1* Het rabbits, compared with those in the WT rabbits (Fig. 3A). IHC and oxytalan staining showed the presence of zonules in the *FBN1* Het rabbits, while reduced fibrillin-1 was observed by IHC in zonules of the *FBN1* Het rabbits (Fig. 3B). Additionally, Hematoxylin and Eosin (H&E) staining and statistical analysis showed significantly decreased corneal epithelium and nonpigmented epithelium of the ciliary body in the *FBN1* Het rabbits compared with those in WT rabbits (Fig. 3C-F) (*P*<0.01) (Hiraoka et al., 2013). These results suggest that the *FBN1* Het rabbits faithfully replicate the eye symptoms of MPL syndrome.

### Aortic dilatation in *FBN1* Het rabbits

**Fig. 4.**
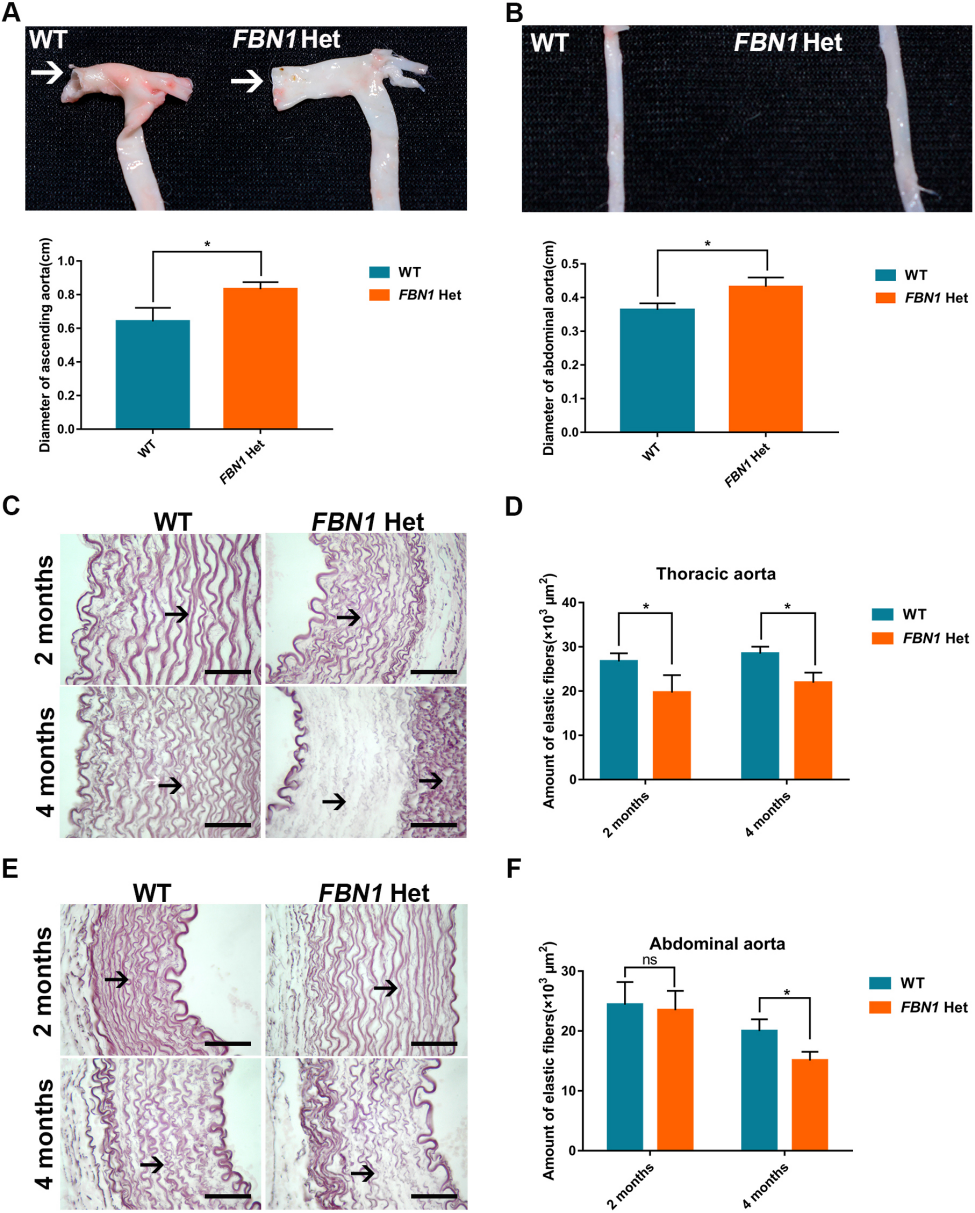
**Aortic dilatation and elastic fiber degradation in *FBN1* Het rabbits.** (A) Aortic dilatation and decrease of elasticity in the ascending aorta (arrows), and the aortic arch of *FBN1* Het rabbits. (B) Aortic dilatation in the abdominal aorta of *FBN1* Het rabbits. (C) Weigert stain showing fracture and age-related decrease in elastic fibers (arrows) in the thoracic aorta in *FBN1* Het rabbits from 2 to 4 months of age. (D) Statistical analysis showing a decrease in elastic fibers in the thoracic aorta in *FBN1* Het rabbits at 2 and 4 months of age. (E) Weigert stain showing age-related decrease in elastic fibers (arrows) in the abdominal aorta in *FBN1* Het rabbits from 2 to 4 months of age. (F) Statistical analysis showing decrease in elastic fibers in the abdominal aorta in *FBN1* Het rabbits at 2 and 4 months of age. Data are presented as mean±s.e.m. and analyzed using Student's *t*-tests with GraphPad Prism software 6.0. *P<0.05. *n*=6. Scale bars: 50 μm.

Aortic dilatation is the main clinical feature of MPL syndrome (Passarge et al., 2016). Moreover, it was previously reported that decreased aortic elasticity is observed in children with *FBN1* mutations (Akazawa et al., 2016). To examine the phenotype of the aorta in the *FBN1* Het rabbits, the aorta was isolated, and histopathology and morphological analyses were performed at the ages of 2 and 4 months. The results showed the decreased elasticity, flat ascending aorta in the *FBN1* Het rabbits, compared with the cylindrical, normal ascending aorta in WT controls (Fig. 4A). Moreover, dilatation in the ascending aorta and abdominal aorta was observed in the *FBN1* Het rabbits, but not in WT controls (Fig. 4A,B) (*P*<0.05). Weigert staining and statistical analysis confirmed an age-related decrease in elastic fibers in thoracic and abdominal aortas of the *FBN1* Het rabbits (Fig. 4C-F) (*P*<0.05). These results suggest that the *FBN1* Het rabbits faithfully reproduce the aortic symptoms of MPL syndrome.

### Skin symptoms and muscle wasting in *FBN1* Het rabbits

**Fig. 5.**
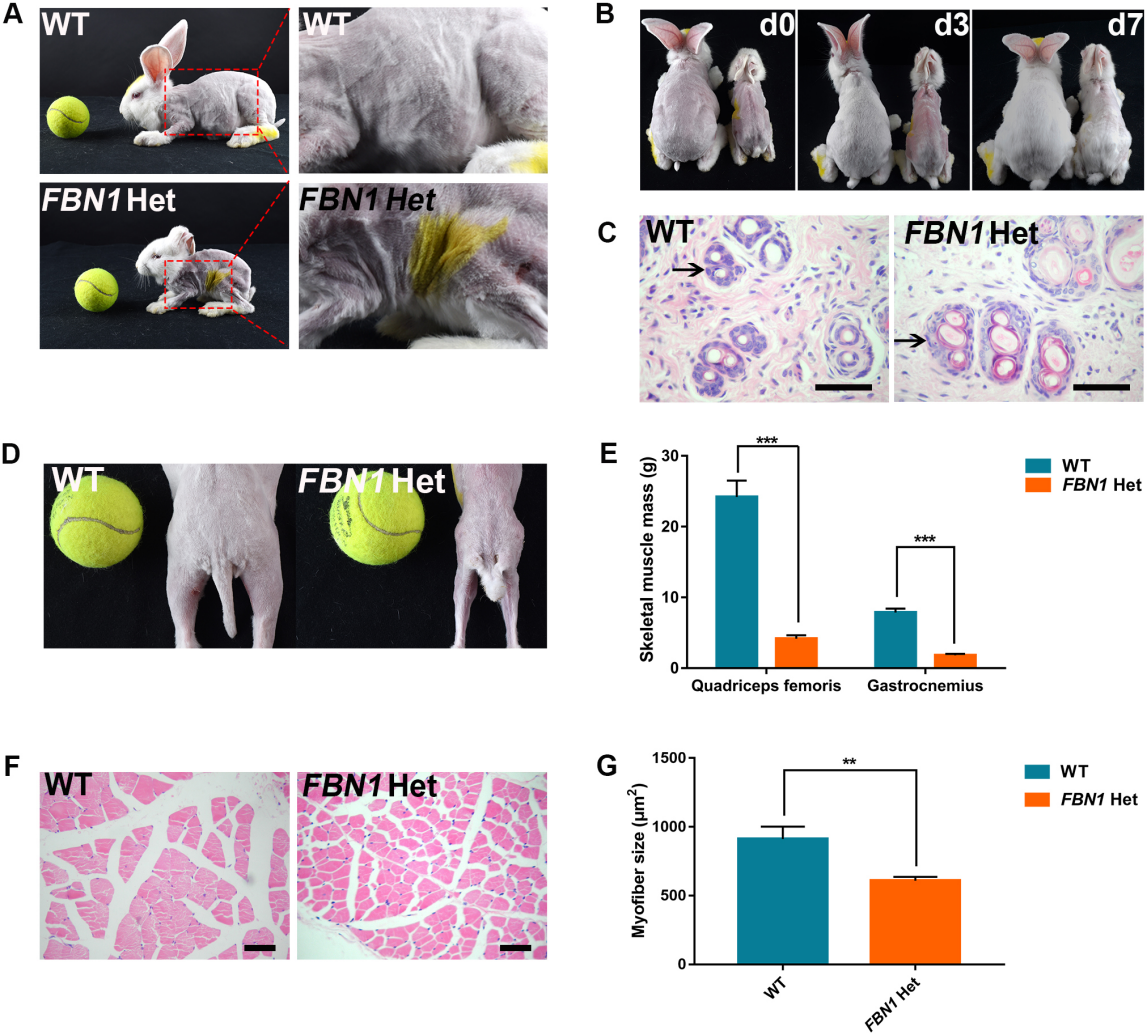
**Skin symptoms and muscle wasting in *FBN1* Het rabbits.** (A) Photograph showing atrophic and loose skin in the *FBN1* Het rabbits. (B) Photograph showing slower speed of hair re-growth in the *FBN1* Het rabbits compared with WT controls (left, WT control; right, *FBN1* Het rabbit). ‘d0’ represents 0 day after shaving hair; ‘d3’ represents 3 days after shaving hair; ‘d7’ represents 7 days after shaving hair. (C) H&E stain showing the degenerated hair follicles (arrow) of *FBN1* Het rabbits (*n*=6). (D) Photograph showing the slender skeletal muscles of *FBN1* Het rabbits. (E) Weight comparison results show the significantly reduced gastrocnemius and quadriceps sizes in *FBN1* Het rabbits. *n*=6. (F) H&E staining and statistics show significantly slender myofibers in *FBN1* Het rabbits. (G) Statistical analysis showing thinner myofibers in *FBN1* Het rabbits compared with those in WT controls. *n*=30. Data are presented as mean±s.e.m. and analyzed using Student's *t*-tests with GraphPad Prism software 6.0. **P<0.01; *** *P*<0.001. Scale bars: 50 μm.

Previous studies have reported that loose skin and sparse scalp hair contribute to progeroid symptoms in patients with MPL syndrome (Jacquinet et al., 2014). To examine skin symptoms in *FBN1* Het rabbits, the appearance and histology of the skin was recorded in the WT and *FBN1* Het rabbits. As shown in Fig. 5A and B, atrophic, loose skin and slower hair growth were observed in the *FBN1* Het rabbits compared with the WT rabbits. The *FBN1* Het rabbits also displayed degenerated hair follicles, particularly in the outer root sheath, which were different from the compact hair follicles and outer root sheath observed in WT rabbits (Fig. 5C).

Reduced muscle mass occurs in patients with mutations of the *FBN1* gene (Behan et al., 2003; Haine et al., 2015; Jacquinet et al., 2014). Furthermore, a more severe musculoskeletal phenotype is found in patients with *FBN1* premature termination codon (PTC) mutation than in patients with the in-frame mutation (Haine et al., 2015). In this study, skeletal muscle wasting was also observed in the *FBN1* Het rabbits (Fig. 5D). Weight comparison results confirmed the significantly reduced gastrocnemius and quadriceps in the *FBN1* Het rabbits (Fig. 5E). The results of H&E staining and statistical analysis showed significantly thinner myofibers in the *FBN1* Het rabbits compared with those in WT controls (Fig. 5F,G) (*P*<0.01). These results suggest muscle wasting in the *FBN1* Het rabbits.

### Severely damaged ear cartilage and retarded growth of *FBN1* Het rabbits

**Fig. 6.**
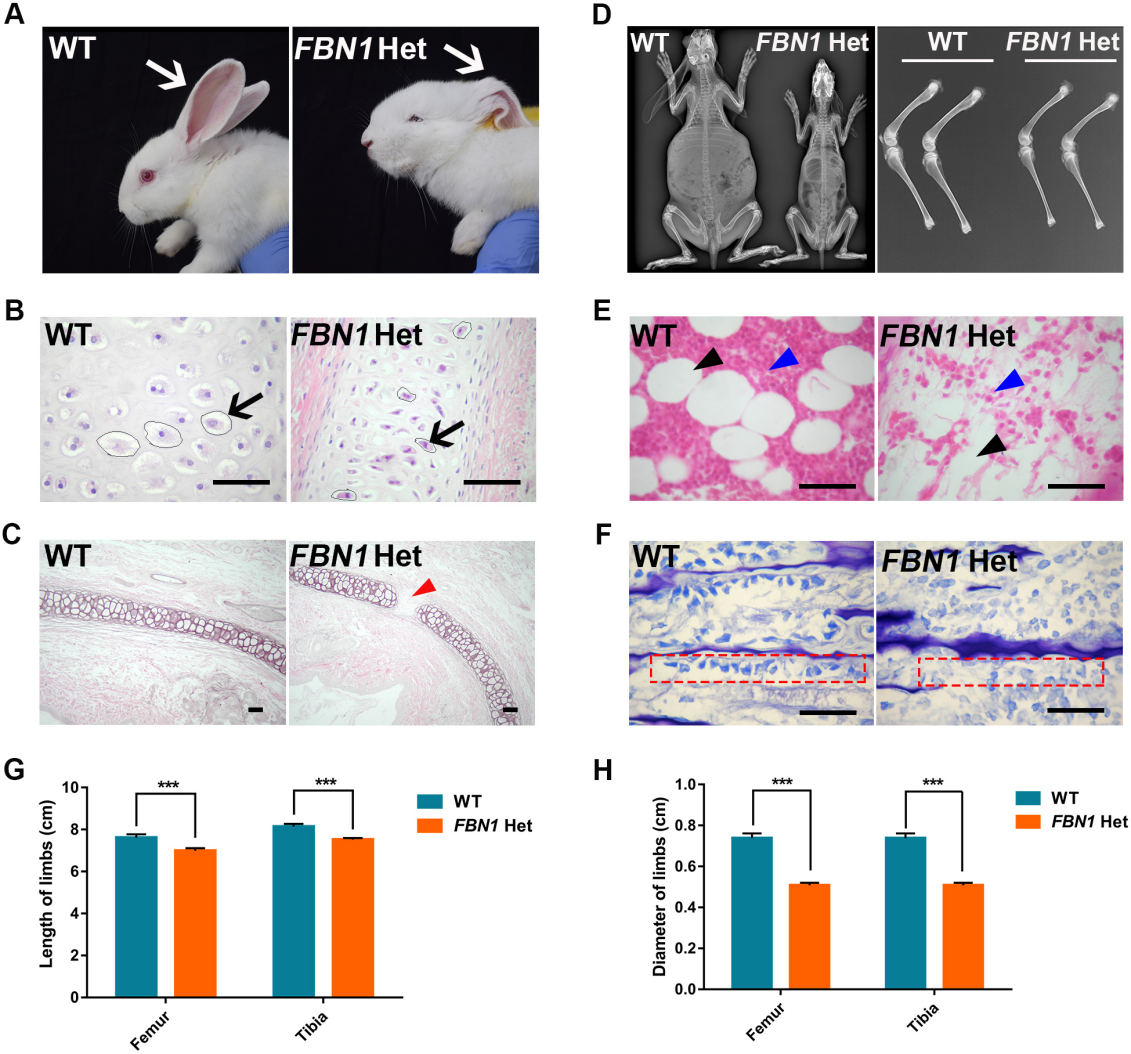
**Severely damaged ear cartilage and retarded growth in *FBN1* Het rabbits.** (A) Photograph showing the bent ears of *FBN1* Het rabbits. (B) H&E stain showing the atrophy of ear cartilage cells (arrow) in *FBN1* Het rabbits. (C) Weigert stain showing the fractured ear elastic cartilage (red arrow) in *FBN1* Het rabbits. (D) X-ray detection showing the shorter and thinner femurs and tibiae of *FBN1* Het rabbits compared with those of WT controls. (E) H&E stain showing the paucity of marrow cells (blue arrowhead) and bone marrow adipocytes (black arrowhead) in *FBN1* Het rabbits compared with WT controls. (F) Toluidine Blue staining showing dissociative and sparse osteoblasts (red dashed line box) in *FBN1* Het rabbits. (G) Statistical comparison of the length of limbs between the *FBN1* Het rabbits and WT controls. (H) Statistical comparison of the diameter of limbs (narrow diaphysis part) between the *FBN1* Het rabbits and WT controls. Data are presented as mean±s.e.m. and analyzed using Student's *t*-tests with GraphPad Prism software 6.0. ***P<0.001. *n*=6. Scale bars: 50 μm.

Previous studies have demonstrated the incidence of low-set ears, caused by cartilage damage, in patients with MPL syndrome (Takenouchi et al., 2013). In this study, compared with the upright and flexible ears of WT rabbits, bent ears were observed in the *FBN1* Het rabbits (Fig. 6A). Furthermore, H&E and Weigert staining showed atrophy or necrosis of the chondrocytes (Fig. 6B) and inconsecutive ear cartilage (Fig. 6C) in the *FBN1* Het rabbits.

Early growth retardation and bone impairment have been widely reported in patients with MPL syndrome (Jacquinet et al., 2014). In order to investigate the influence of truncation of *FBN1* C-terminus on skeletal development, X-ray assay, histological and statistical analyses were carried out, comparing the *FBN1* Het and WT rabbits. As shown in Fig. 6D, significantly smaller body and shorter and thinner limbs were observed in the *FBN1* Het rabbits compared with the WT controls. Statistical analysis revealed the significantly decreased length and diameter of femurs and tibiae in the *FBN1* Het rabbits compared with those in the WT controls (Fig. 6G,H) (*P*<0.001). In addition, the results of H&E staining showed a paucity of marrow cells and bone marrow adipocytes in the *FBN1* Het rabbits compared with the WT controls (Fig. 6E). Furthermore, Toluidine Blue staining revealed significantly decreased osteoblasts in the zone of bone deposition in the *FBN1* Het rabbits compared with WT controls (Fig. 6F).

### Congenital lipodystrophy of *FBN1* Het rabbits

**Fig. 7.**
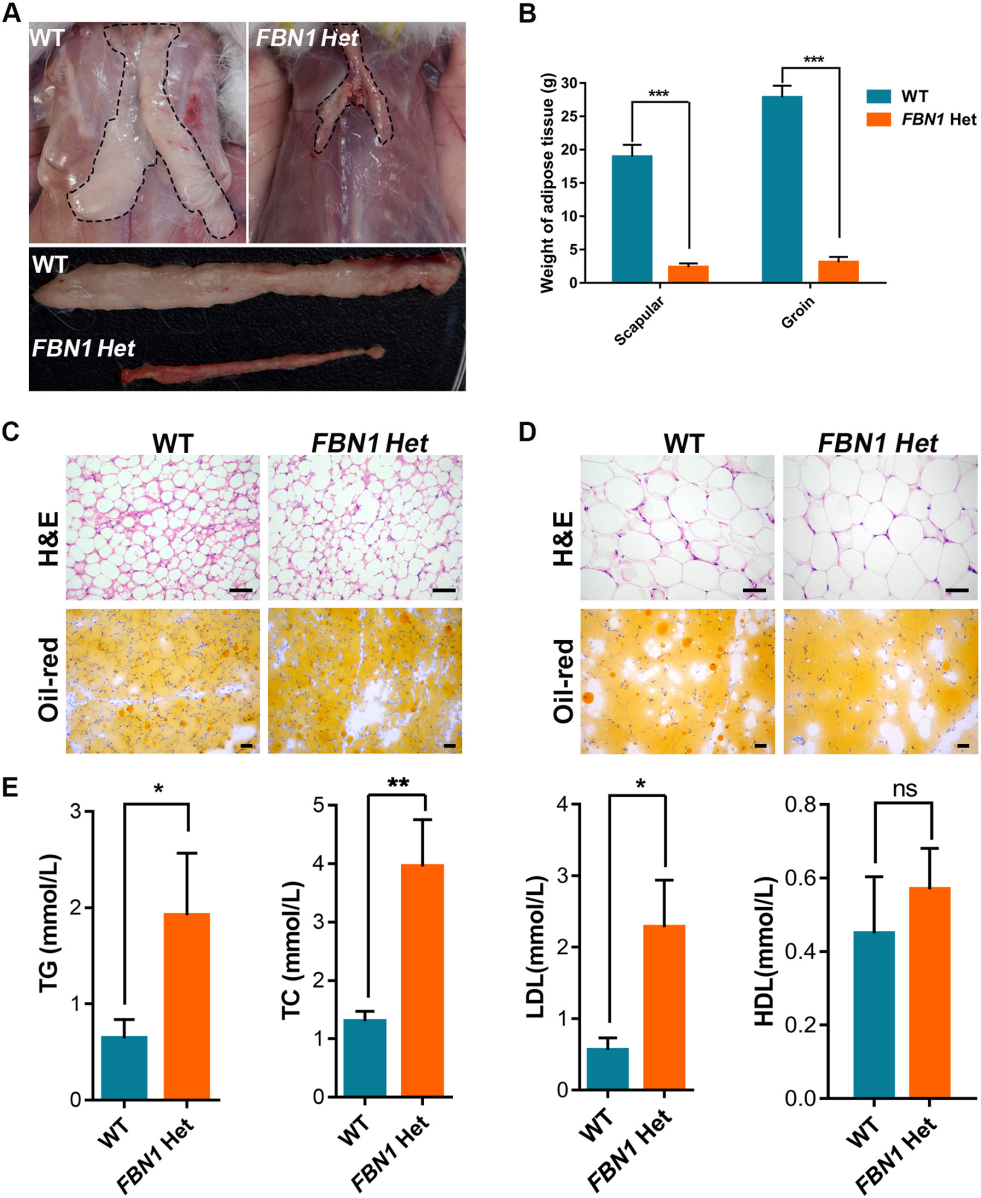
**Congenital lipodystrophy in *FBN1* Het rabbits.** (A) Anatomy showing dysplasia of adipose tissue in *FBN1* Het rabbits. (B) Comparison of adipose tissue weights, showing significantly reduced adipose tissue in *FBN1* Het rabbits. (C) H&E (paraffin section) and Oil Red O (frozen section) staining of brown adipose tissue of *FBN1* Het rabbits and WT rabbits. (D) H&E (paraffin section) and Oil Red O (frozen section) staining of white adipose tissue of *FBN1* Het rabbits and WT rabbits. (E) Increased triglyceride (TG), cholesterol (TC), low density lipoprotein (LDL) and high density lipoprotein (HDL) levels in *FBN1* Het rabbits compared with those in WT rabbits. Data are presented as mean±s.e.m. and analyzed using Student's *t*-tests with GraphPad Prism software 6.0. *P<0.05; **P<0.01; ***P<0.001; ns, not significant. *n*=6. Scale bars: 50 μm.

Severe congenital lipodystrophy, one of the hallmarks of MPL syndrome, has been reported in clinical cases and mouse models (Duerrschmid et al., 2017; Passarge et al., 2016). A highly conserved region of the C-terminus of *FBN1* was speculated to be related to congenital lipodystrophy in humans (Davis et al., 2016). In order to determine the phenotypes of congenital lipodystrophy in *FBN1* Het rabbits, adipose tissues (scapular and groin) were isolated and used for weight comparisons, H&E staining and Oil Red O staining. The results showed significantly decreased adipose tissues in the *FBN1* Het rabbits compared with the WT controls (Fig. 7A,B) (*P*<0.001). H&E and Oil Red O staining showed no significant pathologic changes in brown and white adipose tissues in the *FBN1* Het rabbits (Fig. 7C,D). The serum assay showed significantly increased triglycerides (TG), total cholesterol (TC) and low-density lipoprotein (LDL) levels in the *FBN1* Het rabbits compared with those in the WT controls (Fig. 7E) (*P*<0.05). These results confirm that the PTC mutation in the highly conserved region of C-terminus of *FBN1* induces lipodystrophy in rabbits (Jacquinet et al., 2014).

### Abnormal glucose metabolism of *FBN1* Het rabbits

**Fig. 8.**
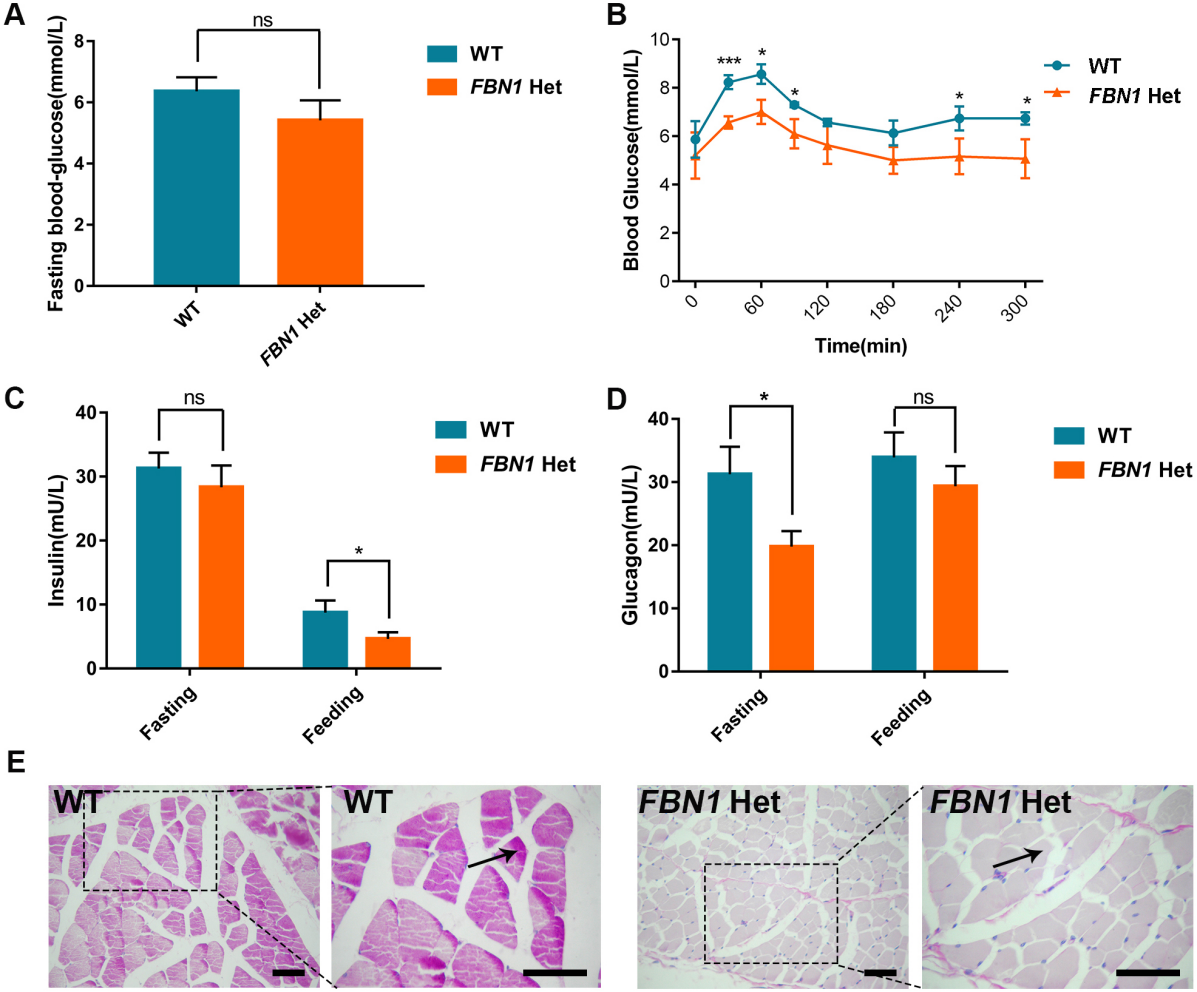
**Abnormal glucose metabolism in *FBN1* Het rabbits.** (A) Fasting blood-glucose (FBG) showing maintained euglycemia in the *FBN1* Het rabbits compared with the WT controls after 12 h fasting. (B) Glucose tolerance test (GTT) showing that *FBN1* Het rabbits have decreased ability to metabolize blood glucose compared with WT controls. (C) Serum ELISA assay showing lower insulin levels in *FBN1* Het rabbits after feeding. (D) Serum ELISA assay showing lower glucagon levels in *FBN1* Het rabbits after fasting. (E) PAS stain showing defect of glycogen deposition (arrow) in the skeletal muscle of *FBN1* Het rabbits. Data are presented as mean±s.e.m. and analyzed using Student's *t*-tests with GraphPad Prism software 6.0. *P<0.05; ***P<0.001. *n*=6. Scale bars: 50 μm.

Recent studies have reported that the C-terminal cleavage product of *FBN1*, a fasting-induced protein hormone (asprosin) functioning in the activation of the G protein-cAMP-PKA pathway, results in rapid glucose release into the circulation (Romere et al., 2016). To confirm the function of asprosin in glucose metabolism, fasting blood glucose (FBG), serum insulin and glucagon levels were examined, and glucose tolerance tests (GTTs) were performed, in the *FBN1* Het and WT rabbits. The results of the FBG tests showed maintained euglycemia in the *FBN1* Het rabbits compared with the WT controls (Fig. 8A). However, GTTs showed a deficiency in glucose uptake in the *FBN1* Het rabbits compared with the WT controls (Fig. 8B). In addition, serum insulin and glucagon levels in the *FBN1* Het rabbits were lower than those in WT controls after fasting and feeding (Fig. 8C,D). Furthermore, Periodic Acid-Schiff (PAS) staining showed deficiency of glycogenesis in the skeletal muscles of the *FBN1* Het rabbits (Fig. 8E). These results confirm that asprosin plays a role in glucose uptake, hormonal regulation and glycogenesis in this animal model.

## DISCUSSION

In this study, *FBN1* Het rabbits were generated by the CRISPR/Cas9 system with high efficiency, and we identified a crucial role for *FBN1* in the assembly of the extracellular matrix and microfibrils (Jensen and Handford, 2016). The phenotypes of aortic dilatation were found in the *FBN1* Het rabbits. Owing to a truncated C-terminus of fibrillin-1, new phenotypes of lipodystrophy and progeroid appearance were also seen in the MPL rabbit model. Furthermore, the prominent features of curved ears helped to show that *FBN1* Het rabbits had damaged cartilage. However, the *FBN1* Het rabbits with short stature failed to recapitulate the hyperextensible joint seen in human cases. Therefore, this novel rabbit model could be used as a model for recapitulating MPL syndrome in humans.

As expected, a high mortality rate was seen in the F0 *FBN1* mutant rabbits. Fortunately, the F0-7 male rabbits could live to sexual maturity and were used for mating with the WT rabbits. The mosaicism in the F0-7 rabbits indicated that germline and genotype chimeras generated by CRISPR/Cas9 could significantly expand studies of unknown gene functions in cells or animals (Wang et al., 2017). We could not obtain homozygous gene knockout rabbits owing to the high mortality rate of the *FBN1* Het rabbits. Further treatment and animal breeding are needed for the generation of double-allele knockout rabbits in future studies.

Fibrillin-1 assembles to form microfibrils in the extracellular matrix, which are essential for elasticity in all tissues (Baldock et al., 2006). However, the disrupted mechanisms of microfibril organization and assembly are still unclear in MPL syndrome. Experiments *in vitro* show that the secretion of full-length fibrillin-1 from cells is mediated by its C-terminus (Jensen et al., 2014). Our results confirmed reduced secretion of the truncated C-terminus of fibrillin-1 and reduced elastin in the extracellular matrix. Moreover, reduced mRNA expression levels were observed in patients with PTC mutations in *FBN1*, which were associated with NMD (Aubart et al., 2015*;* Schrijver et al., 2002). Therefore, the *FBN1* Het rabbit model could be used for mechanistic studies of microfibril organization and preclinical research on MPL syndrome.

Recently, the new hormone asprosin, which is cleaved from the C-terminus of fibrillin-1 and functions in glucose metabolism, has been reported in patients with MPL syndrome and *Fbn1^NPS/+^* mice (Duerrschmid et al., 2017; Romere et al., 2016). Both the patients and *Fbn1^NPS/+^* mice exhibited lipodystrophy. Our results also confirmed a significant decrease in blood glucose levels and lipodystrophy in the *FBN1* Het rabbits. Previous studies have demonstrated that mutation of the C-terminal peptide of *FBN1* (asprosin) failed to differentiate adipocytes, which was thought to be related to congenital lipodystrophy in MPL syndrome (Davis et al., 2016). In addition, congenital lipodystrophy and neonatal progeroid were frequently reported in the patients with MPL syndrome. This MPL syndrome was caused by frameshift mutation or exon 65 skipping of *FBN1*, inducing disruption of the C-terminal furin cleavage site or asprosin in the clinic (Jacquinet et al., 2014). Therefore, whether a mutation in the asprosin coding sequence rather than the C-terminal furin cleavage site causes hypoglycemia and lipodystrophy in MPL syndrome still needs to be shown experimentally.

In this study, we mutated the C-terminus of the rabbit fibrillin-1 protein and generated stable genetically heterozygous offspring of *FBN1* Het rabbits. These rabbits showed classical features of MPL syndrome, including mild signs of MFS, lipodystrophy, muscle wasting and glucose metabolic disorders. This rabbit model can be helpful for understanding the pathogenesis of MPL syndrome and screening new drugs or therapies for its treatment.

## MATERIALS AND METHODS

### Ethics statement

New Zealand rabbits (*Oryctolagus cuniculus*) were maintained at the Laboratory Animal Center of Jilin University. All experiments involving rabbits in this study were performed in accordance with the Animal Care and Use Committee guide from Jilin University.

### CRISPR/Cas9-mediated rabbit *FBN1* gene editing

The sgRNAs targeting the C-terminus of fibrillin-1 were designed, assembled and *in vitro* transcribed as previously described (Sui et al., 2016). The procedures for microinjection and embryo transfer were as previously described (Yan et al., 2014). Briefly, female New Zealand White rabbits of 6-8 months of age were superovulated with 50 IU follicle stimulating hormone (FSH) at intervals of 12 h for six rounds, then mated with male rabbits, and injected with 100 IU human chorionic gonadotropin (HCG). The female rabbits were then euthanized and their oviducts were flushed with 5 ml Dulbecco's phosphate buffered saline/bovine serum albumin (DPBS-BSA) for the collection of zygotes. Rabbit embryos in the pronuclear stage were collected. A mixture containing Cas9 mRNA (200 ng/µl) and sgRNA (30 ng/µl) was microinjected into the cytoplasm of zygotes. The injected embryos were transferred into Earle's balanced salt solution (EBSS) medium and cultured at 38.5°C with 5% CO_2_ for 1 h. Approximately 40-60 injected zygotes were transferred into the oviduct of each recipient rabbit.

### Mutation detection and off-target analysis

Mutation detection was performed as previously described (Yuan et al., 2016). Genomic DNA from *FBN1* Het rabbits and WT controls was extracted from a small piece of ear tissue using the TIANamp Genomic DNA Kit (Tiangen, China) according to the manufacturer's instructions. PCR primers for the sgRNA target sites are listed in Table S3. PCR products were gel extracted and cloned into pGM-T vector (Tiangen). At least ten positive plasmid clones were sequenced and analyzed using BLAST (NCBI; https://blast.ncbi.nlm.nih.gov/Blast.cgi) and DNAMAN (https://www.lynnon.com/pc/framepc.html).

Potential off-target sequences (POTS) were predicted using the CRISPR Design Tool (http://crispr.mit.edu/). The PCR products were subjected to T7EI assay (Guschin et al., 2010) and Sanger sequencing. Primers for these POTS are listed in Table S4.

### Real-time quantitative PCR

Total RNA from the ear skin of *FBN1* Het and WT rabbits was extracted using TRNzol-A reagent (Tiangen) according to the manufacturer's instructions and reverse-transcribed to cDNA using a FastQuant RT Kit (with gDNase) (Tiangen). Primers for quantitative PCR (qPCR) are listed in Table S3. qPCR was performed using an ABI PRISM 7500 system (Applied Biosystems, USA). The 2^−ΔΔCT^ method was used to determine *FBN1* gene expression, which was normalized to the amount of *GAPDH* mRNA. All experiments were repeated three times for each gene, and data are expressed as mean±s.e.m.

### Histopathology

*FBN1* Het rabbits and WT controls were euthanized by injection with pentobarbital sodium. The lungs, eyes, aorta, quadriceps, thigh skin, ears and adipose tissue were fixed with 4% paraformaldehyde for 24 h and processed for paraffin embedding before sectioning. The tibiae and knee joints were subsequently decalcified in 15% EDTA for 2 weeks, followed by dehydration in increasing concentrations of ethanol (70% for 6 h, 80% for 1 h, 96% for 1 h and 100% for 3 h). The tibiae and knee joints were then washed with xylene and embedded in paraffin (Sui et al., 2016). Slides were prepared using 6 μm sections and stained with H&E, Weigert stain (for elastic fibers), PAS (for muscle glycogen), oxytalan stain (for zonules) or Toluidine Blue (for osteoblasts). IHC (11C1.3, Abcam) was performed to identify zonules (Davis et al., 2016; Inoue et al., 2012; Lima et al., 2010; Yang et al., 2014). Oil Red O staining of adipose tissue was performed on frozen sections using standard protocols (Davis et al., 2016).

### Secretion assay

Rabbit skin fibroblasts were isolated as previously described (Rittie and Fisher, 2005). Detection of fibrillin-1 secretion was performed as previously described (Jensen et al., 2014). Briefly, skin that had been isolated from newborn WT or *FBN1* Het rabbits was cut into 0.5 mm^3^ pieces. The pieces were then digested with 1 mg/ml type I collagen solution (Sigma-Aldrich) for 3 h. Skin fibroblasts were transferred to 24-well plates and grown for a further 3 days in Dulbecco's modified Eagle medium (DMEM) with 10% serum. Then, immunofluorescence was performed as previously described (Jensen et al., 2014). Anti-fibrillin-1 (1:100) (11C1.3, Abcam) was used as the primary antibody for immunofluorescence.

### Western blotting

Western blotting was performed as previously described (Wipff et al., 2010). The fibroblasts were homogenized in radioimmunoprecipitation assay (RIPA) buffer and protein concentrations measured using bicinchoninic acid assay (BCA) according to the manufacturer's instructions (Byotime, China). Samples containing equal amounts of protein were loaded and run on 10% polyacrylamide-SDS gels, followed by immunoblotting onto a nitrocellulose membrane (BOSTER, China). Membranes were incubated overnight at 4°C with anti-fibrillin-1 (1:500) (11C1.3, Abcam) as a primary antibody. Membranes were then incubated with a secondary horseradish peroxidase (HRP)-conjugated anti-mouse antibody (A0216, Byotime) and imaged using a BeyoECL Plus kit (P0018, Byotime). A β-actin primary antibody (1:2000; AA128, Byotime) was used as a loading control.

### X-ray detection

X-ray detection was performed as previously described (Sui et al., 2016). X-ray autoradiographic images of whole-body skeletons, tibiae and femurs were taken using a YEMA Radiography System with a digital camera attached (Varian, USA) on X-ray film (ROTANODE, Japan). The images were taken at 40 kV with 3 mAs exposure.

### Transmission electron microscopy

Transmission electron microscopy was performed as previously described (Zhang et al., 2015). The *FBN1* Het rabbits and WT rabbits were euthanized at 4 months of age, and then the aortic root was isolated and fixed in 4% glutaraldehyde for 24 h at 4°C. Before the aortas were embedded in epon 812, samples were rinsed with 1% OsO_4_ and dehydrated in a series of ethanol solutions with increasing concentrations. The ultrathin sections of aortic tunica media were cut with a diamond knife and mounted on copper grids. Elastin and microfibrils were observed under an electron microscope (H-7640, Hitachi, Japan).

### Blood assay

*FBN1* Het and WT rabbits were anesthetized at 1 month of age and heart blood was collected. Serum samples were extracted through precipitation and centrifugation. Triglyceride (TG) levels were detected using a triglyceride test kit (Ri Yuan, China). Total cholesterol (TC) levels were detected using a TC test kit (Ri Yuan). Serum low density lipoprotein (LDL) levels were measured using an LDL test kit (Ri Yuan). Serum high density lipoprotein (HDL) levels were measured using an HDL test kit (Ri Yuan). Heart blood from the *FBN1* Het and WT rabbits was collected after 12 h fasting and 1 h feeding. Serum insulin levels were measured with an insulin ELISA kit (IBL, Germany) following the manufacturer's instructions. Serum glucagon levels were measured with a glucagon ELISA kit (IBL) following the manufacturer's instructions. FBG tests and GTTs were carried out as previously described (Zhang et al., 2013).

### Analyses of body and tissue weights, and survival

Analysis of body weight and survival time was performed as previously described (Sui et al., 2016). The deaths of rabbits, including WT and *FBN1* Het rabbits, were recorded daily and used for the survival analysis. Body weights were recorded weekly.

Gastrocnemius, quadriceps and adipose tissues from the scapular and groin of *FBN1* Het and WT groups were isolated and weighed at 2 months of age. Thirty myofibers of quadricep were used for analyses of cross-sectional area. The diameters and lengths of rabbits’ tibiae and femurs were recorded and analyzed. All data are presented as mean±s.e.m., and at least three individual determinations were used in all experiments. Data were analyzed with the Student's *t*-test using GraphPad Prism software 6.0. *P<0.05 was considered statistically significant.

## Supplementary Material

Supplementary information
