# Supplementary material for: Truncated C-terminus of fibrillin-1 induces Marfanoid-progeroid-lipodystrophy (MPL) syndrome in rabbit
Source: Dis Model Mech. 2018 Apr 9;11(4):dmm031542. doi: 10.1242/dmm.031542 (PMC5963856; doi:10.1242/dmm.031542)
Supplement: Supplementary information [file dmm-11-031542-s1.pdf]

SUPPLEMENTAL FILES

Supplemental figures

Figure S1.

```
#F0-1    TCTGGAATGGGCATGGGCCGAGGAGGCCCGGAGCCACCTGT  //  GTTACGAGTGTAAGATCAATGGCTACCCCAAACGGGGCAGG WT
#F0-2  | TCTGGAATGGGCATGGG------AGGCCCGGAGCCACCTGT  //  GTTACGAGTGTAAGATCAATGGCTACCCCAAAC------GG -12
       | TCTGGAATGGGCATGGGCCGAGGAGGCCCGGAGCCACCTGT  //  GTTACGAGTGTAAGATCAATGGCTACCCCAAAC------GG -6
#F0-3  | TCTGGAATGG-------------------------------  //  ------------------------------------GCAGG -108
       | TCTGGAATGGGCATGGG------------------------  //  -----------------AATG-------------GGGCAGG -95
#F0-4  | TCTGGAATGGGCATGGGCCGAGGAGGCCCGGAGCCACCTGT  //  GTTACGAGTGTAAGATCAATGGCTACCCCAAACGGGGCAGG WT
       | TCTGGAATGGGCATGGG------------------------  //  -----------------------------------GGCAGG -100
#F0-5  | TCTGGAATGGGCATGGGCCGAGGAGGCCCGGAGCCACCTGT  //  GTTACGAGTGTAAGATCAATGGCTACCCCAAACG---CAGG -3
       | TCTGGAATGGGCATGGG------------------------  //  -----------------------------------GGCAGG -100
       | TCTGGAATGGGCATGGGCCGAGGAGGCCCGGAGCCACCTGT  //  GTTACGAGTGTAAGATCAATGGCTACCCCAAAC------GG -6
#F0-6  | TCTGGAATGGGCATGGGCCGAGGAGGCCCGGAGCCACCTGT  //  GTTACGAGTGTAAGATCAATGGCTACCCCAAACGG--CAGG -2
       | TCTGGAATGGGCATGGG--GAGG---CCCGGAGCCACCTGT  //  GTTACGAGTGTAAGATCAATGGCTACCCCAAACGG--CAGG -7
#F0-7  | TCTGGAATGGGCATGG---------------AGCCACCTGT  //  GTTACGAGTGTAAGATCAATGGCTACCCCAAT---GGCAGG -18+1
       | TCTGGAATGGGCATGGGCCGAGGAGGCCCGGAGCCACCTGT  //  GTTACGAGTGTAAGATCAATGGCTACCCCAAACG--GCAGG -2
       | TCTGGAATGGGCATGGGCCGAGGAGGCCCGGAGCCACCTGT  //  GTTACGAGTGTAAGATCAATGGCTACCCCAAACG---GAGG -3
       | TCTGGAATGGGCATGGG------------------------  //  -----------------------------------GGCAGG -100
#F0-8  | TCTGGAATGGGCATGGG------------------------  //  ----------------------------------GGGCAGG -99
       | TCTGGAATGGGCATGGGCCGAGGAGGCCCGGAGCCACCTGT  //  ACGAGTGTAAGATCAATGGCTACCCCAAACGTTTGGGCAGG +3
       | TCTGGAATGGGCATGGG------------------------  //  -----------------------------------GGCAGG -100
#F0-9  | TCTGGAATGG-------------------------------  //  -------------------------------------CAGG -109
       | TCTGGAATGGGCATGGGCCGAGGAGGCCCGGAGCCACCTGT  //  GTTACGAGTGTAAGATCAATGGCTACCCCAAACG--GCAGG -2
#F0-10 | TCTGGAATGGGCATGGGCCGAGGAGGCCCGGAGCCACCTGT  //  GTTACGAGTGTAAGATCAATGGCTACCCCAAACGGG-CAGG -1
       | TCTGGAATGG-------------------------------  //  ------------------------------------GCAGG -108
#F0-11   TCTGGAATGGGCATGGGCCGAGGAGGCCCGGAGCCACCTGT  //  GTTACGAGTGTAAGATCAATGGCTACCCCAAACGGGGCAGG WT
#F0-12 | TCTGGAATGGGCATGGGCC----------------------  //  -----------------------------AAACGGGGCAGG -92
       | TCTGGAATGGGCATGGGCCGAGGAGGCCCGGAGCCACCTGT  //  TAAGATCAATGGCTACCCCAAACGG------------GAA -13
       | TCTGGAATGGGC----CCGGAGGAGGCCCGGAGCCACCTGT  //  GTTACGAGTGTAAGATCAATGGCTACCCCAAAC--GGCAGG -6+1
#F0-13   TCTGGAATGGGCATGGG------------------------  //  -------------------------------ACGGGGCAGG -96
#F0-14   TCTGGAATGGGCATGGGCCGAGGAGGCCCGGAGCCACCTGT  //  GTTACGAGTGTAAGATCAATGGCTACCC-----ACGGCAGG -5+2
#F0-15 | TCTGGAATGGGCATGGGCCGAGGAGGCCCGGAGCCACCTGT  //  GTTACGAGTGTAAGATCAATGGCTACCCCAAACGGGGCAGG WT
       | TCTGGAATGGGCATGGGCCGAGGAGGCCCGGAGCCACCTGT  //  GTTACGAGTGTAAGATCAATGGCTACCCCAAACGG--CAGG -2
#F0-16 | TCTGGAATGGGCATGG-------------------------  //  ---------------------------------------AA -107
       | TCTGGAATGGGCATGGGCCGAGGAGGCCCGGAGCCACCTGT  //  GTTACGAGTGTAAGATCAATGGCTACCCCAAACG---CAGG -3
       | TCTGGAATGGGCATGGGCCGAGGAGGCCCGGAGCCACCTGT  //  GTTACGAGTGTAAGATCAATGGCTACCCCAAACGG--CAGG -2
#F0-17 | TCTGGAATGGGCATGGGCCGAGGAGGCCCGGAGCCACCTGT  //  GTTACGAGTGTAAGATCAATGGCTACCCCAAACGG---AGG -3
       | TCTGGAATGGGCATGGGC------GGCCCGGAGCCACCTGT  //  GTTACGAGTGTAAGATCAATGGCTACCCCAAACGG------ -6
#F0-18 | TCTGGAATGGGCATGGG------------------------  //  ------------------------------AACGGGGCAGG -95
       | TCTGGAATGGGCATGGGCCGAGGAGGCCCGGAGCCACCTGT  //  GTTACGAGTGTAAGATCAATGGCTACCCCAAACG--GCAGG -2
       | TCTGGAATGGGCAT---------------------------  //  ---------------------------------GGGGCAGG -101
       | TCTGGAATGGGCATGGGCCGAGGAGGCCCGGAGCCACCTGT  //  GTTACGAGTGTAAGATCAATGGCTACCCCAAAC------GG -6
#F0-19 | TCTGGAATGGGCATGGGCCGAGGAGGCCCGGAGCCACCTGT  //  GTTACGAGTGTAAGATCAATGGCTACCCCAAACGG--CAGG -2
       | TCTGGAATGGGCATGGGCCGAGGAGGCCCGGAGCCACCTGT  //  GTTACGAGTGTAAGATCAATGGCTACCCCAAACGGGGCA-- -35
       | TCTGGAATGGGCATGGGCCGAGGAGGCCCGGAGCCACCTGT  //  GTTACGAGTGTAAGATCAATGGCTACCCCAAACGGGGCAGG WT
       | TCTGGAATGGGCATGGGCCGAGGAGGCCCGGAGCCACCTGT  //  AGTGTAAGATCAATGGCTACCCCAAACGGAAGCGGAGGAGG +9
#F0-20 | TCTGGAATGGGCAT---------------------------  //  -----------------------------------GGCAGG -103
       | TCTGGAATGGGCATGGG------------------------  //  -------------------------------------TAGG -103+1
#F0-21 | TCTGGAATGGGCATGGGCCGAGGAGGCCCGGGGCCACCTGT  //  TACGAGTGTAAGATCAATGGCTACCCCAAACGGAATGCAGG -1+3
       | TCTGGAATGGGCATGGGC-----------------------  //  --------------------------------------AGG -102
       | TCTGGAATGGGCATGGGCCGAGGAGGCCCGGAGCCACCTGT  //  GTTACGAGTGTAAGATCAATGGCTACCCCAAACG--GCAGG -2
#F0-22 | TCTGGAATGGGCATGGGCCGAGGAGGCCCGGAGCCACCTGT  //  GTTACGAGTGTAAGATCAATGGCTACCCCAAACGGG-CAGG -1
       | TCTGGAATGGGCA----------------------------  //  ---------------------------------------GG -108
#F0-23   TC---------------------------------------  //  ------------------------------------GCAGG -116
#F0-24 | TCTGGAATGGGCATGGG------------------------  //  ------------------------------------GCAGG -101
       | TCTGGAATGGGCATG--------------------------  //  ------------------------------------GTAGG -103+2
#F0-25 | TCTGGAATGGGCATGGG------------------------  //  ------------------------------------GCAGG -101
       | TCTGGAATGGGCATGG-CCGAGGAGGCCCGGAGCCACCTGT  //  GTTACGAGTGTAAGATCAATGGCTACCCCAAACGGGGCAGG -1
       | TCTGGAATGGGCATGGGCCGAGGAGGCCCGGAGCCACCTGT  //  GTTACGAGTGTAAGATCAATGGCTACCCCAAACG--GCAGG -2
#F0-26 | TCTGGAATGGGCATGGGCCGAGGAGGCCCGGAGCCACCTGT  //  GTTACGAGTGTAAGATCAATGGCTACCCCAAACGGGGCAGG WT
       | TCTGGAATGGGCATGGG------------------------  //  ----------------------------------------- -106
#F0-27   TCTGGAATGGGCATGGG------------------------  //  -----------------------------AAACGGGGCAGG -94
#F0-28 | TCTGGAATGGGCATGGG------AGGCCCGGAGCCACCTGT  //  GTTACGAGTGTAAGATCAATGGCTACCCCAAACGGG-CAGG -7
       | TCTGGAATGGGCATGGG---AGGAGGCCCGGAGCCACCTGT  //  GTTACGAGTGTAAGATCAATGGCTACCCCAAACGGG-CAGG -4
```

Disease Models & Mechanisms • Supplementary information

Figure S1. Generation of *FBN1* knockout F0 rabbits.

(A) Sanger sequencing of the targeted region of *FBN1* in F0 rabbits. The highlights of sgRNA sequences and PAM sites are green and red, respectively; Bases of indel are in black within sgRNA sequence; WT represents wild-type *FBN1* sequence was detected.

Figure S2.

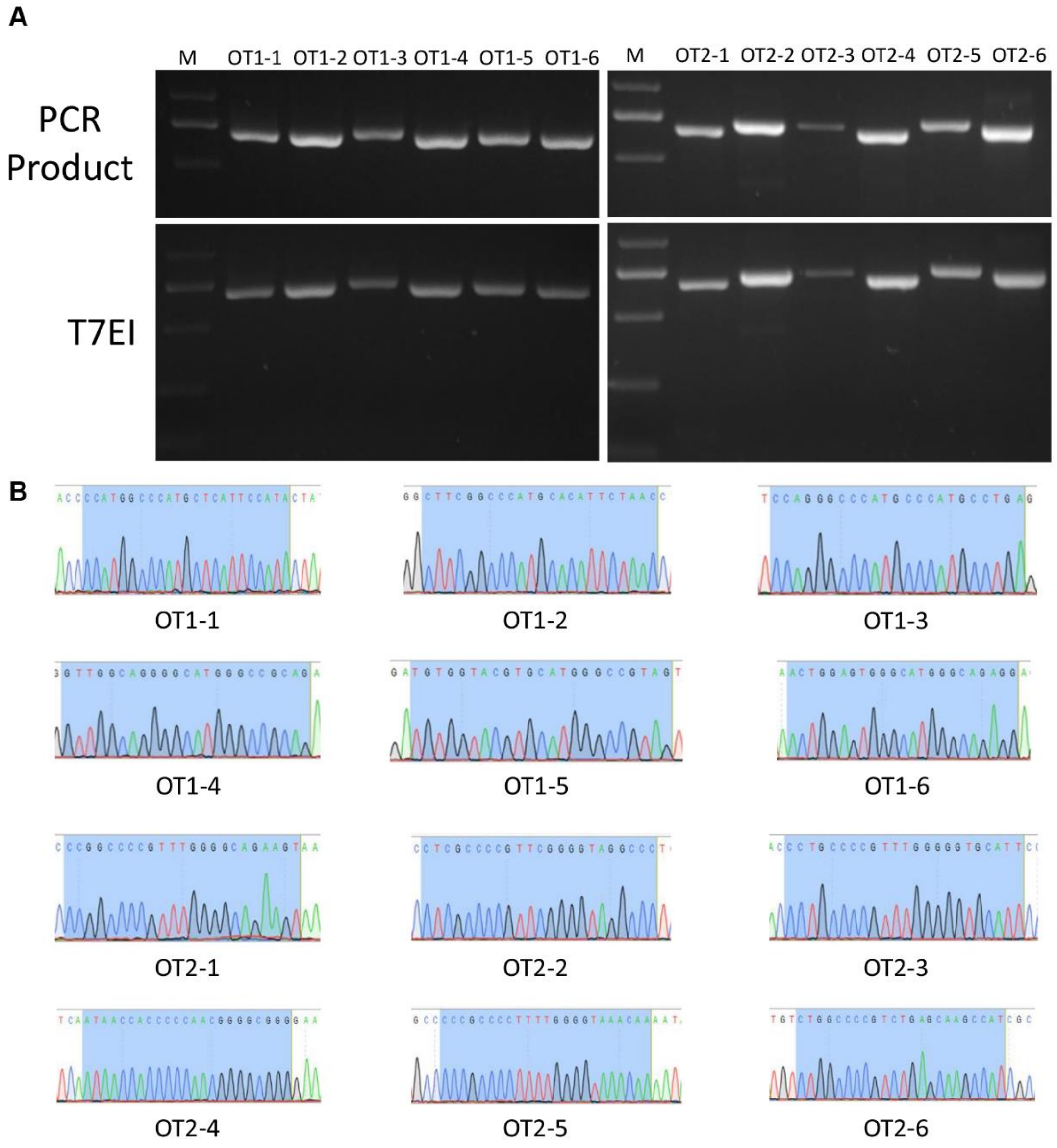

Figure S2. Off-target detection of *FBN1* KO rabbits.

(A) T7E1 analysis of POTS for sgRNA1 and sgRNA2. M, DL2000; OT1represent POTS of sgRNA1; OT2 represent POTS of sgRNA2. (B) Chromatogram sequence analysis of 12 POTS for sgRNA1 and sgRNA2 using PCR products. The sequences of the POTS and the PAM are

Disease Models & Mechanisms • Supplementary information

represented in shadow.

Figure S3.

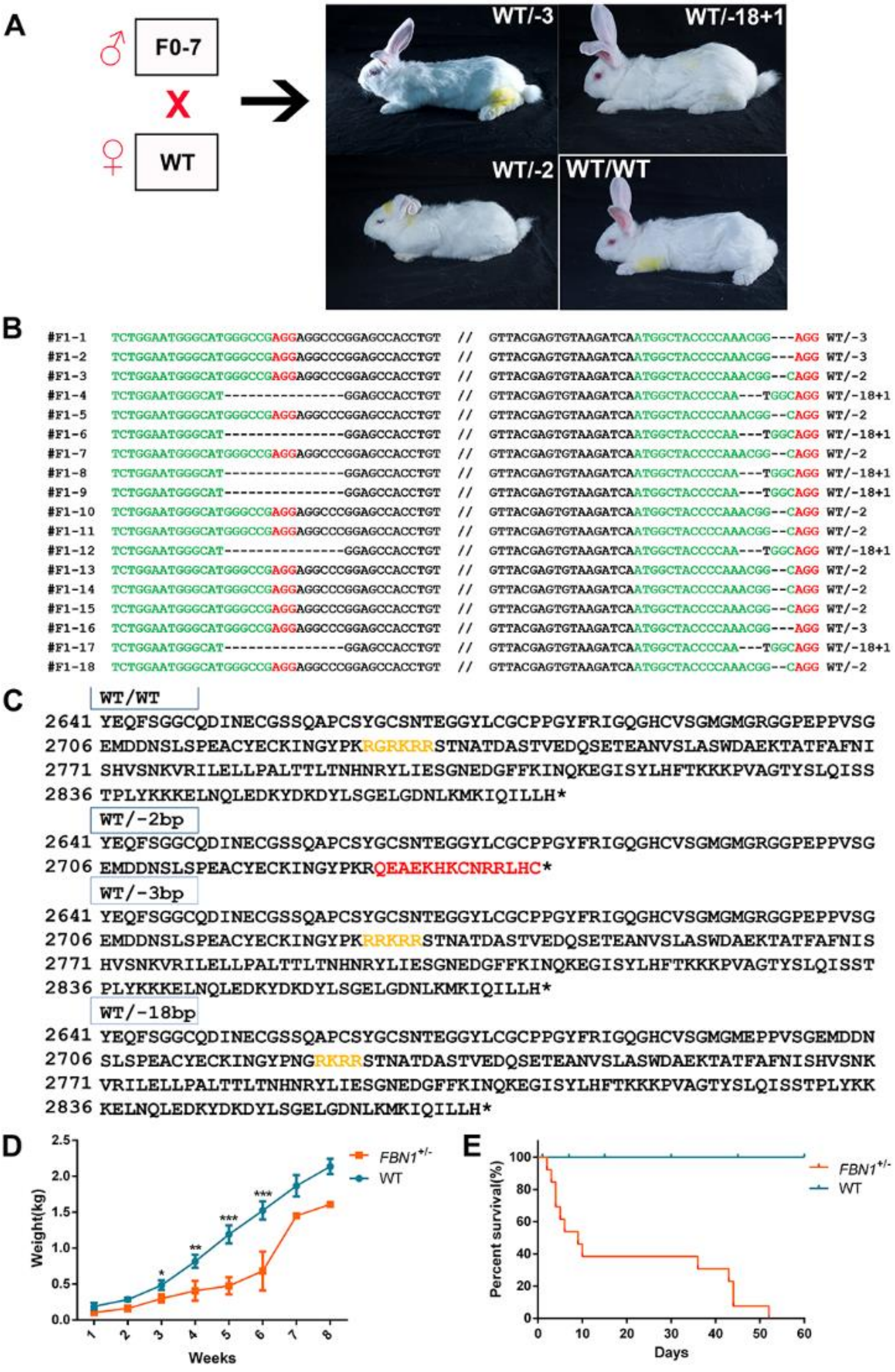

Disease Models & Mechanisms • Supplementary information

Figure S3. Hereditary of *FBN1* KO rabbits.

(A) Generation of F1 rabbits by #F0-7 mated with WT rabbits. (B) T-cloning sequence analysis of the *FBN1* Het rabbits. Target sites of the two sgRNA sequences, sgRNA1 and sgRNA2, are highlighted in green, protospacer-adjacent motif (PAM) sequence is highlighted in red; WT represents gene sequence of rabbit. (C) The predicted protein sequences of *FBN1* Het rabbits, showing the premature termination codon (PTC) mutation in *FBN1*. (D) The weight comparation shows the growth retarded were determined in the *FBN1* Het rabbits (n=12). (E) Survival curves shows the high mortality of *FBN1* Het rabbits (WT, n=6; *FBN1* Het, n=12). Data are presented as mean ± SEM and analyzed using student's t-tests with Graphpad Prism software 6.0. *p < 0.05; ** p < 0.01; *** p < 0.001. Scale bars: 50 µm.

Supplemental tables

Table S1: Generation of *FBN1* knockout rabbits using CRISPR/Cas9

Recipient	gRNA/Cas9 (ng/µl)	No. embryos transferred	No. newborns (%)	No. mutant rabbits (%)
1	30/200	56	3(5)	2(67)
2	30/200	56	10(18)	9(90)
3	30/200	42	10(24)	10(100)
4	30/200	43	5(12)	5(100)
Total (average)		197	28(14)	26(93)

Table S2: Genotype and phenotype in F1 generation rabbits

NO.F1 generation	Genotype	Transcription is terminated prematurely or not	Phenotype
#1-1	WT/-100	√	√
#1-2	WT/-3	×	×
#1-3	WT/-3	×	×
#1-4	WT/-2	√	√
#1-5	WT/-18+1	×	×
#1-6	WT/-2	√	√
#1-7	WT/-18+1	×	×
#1-8	WT/-2	√	√
#2-1	WT/-18+1	×	×
#2-2	WT/-18+1	×	×
#2-3	WT/-2	√	√
#2-4	WT/-2	√	√
#2-5	WT/-18+1	×	×
#2-6	WT/-100	√	√
#2-7	WT/-2	√	√
#3-1	WT/-2	√	√
#3-2	WT/-2	√	√
#3-3	WT/-3	×	×
#3-4	WT/-18+1	×	×
#3-5	WT/-100	√	√
#3-6	WT/-2	√	√

Table S3: Oligonucleotide used for sgRNA, PCR and qPCR.

NO.	Name	Primers	Sequence(5'-3')	Produce size (bp)
**1**	SgRNA1	gRNA-1-F	TAGGTCTGGAATGGGCATGGGCCG	20
		gRNA-1-R	AAACCGGCCCATGCCCATTCCAGA	
**2**	SgRNA2	gRNA-2-F	TAGGATGGCTACCCCAAACGGGGC	20
		gRNA-2-R	AAACGCCCCGTTTGGGGTAGCCAT	
**3**	FBN1	FBN1-F	TGCCTCACATCTAGCTCCCT	745
		FBN1-R	AGAAACTCCAGAAAGCCCCG	
**4**	qPCR-FBN1	FBN1-qPCR-F	TAGCTCCTTCCTGTGGCTCC	90
		FBN1-qPCR-R	GGCATAGACAGTGGTCGTCA	
**5**	qPCR-GAPDH	GAPDH-F	ATCCATTCATTGACCTCCACTAC	179
		GAPDH-R	GTACTGGGCACCAGCATCAC	

1 and 2 were used for the construction of sgRNA expression plasmids; 3 was used for the PCR mutation detection; 4 and 5 were used for the qPCR detection.

Disease Models & Mechanisms • Supplementary information

Table S4. 12 potential off-target sites examined by PCR and primers used for list.

sgRNA	Potential Off Target Site	Number of mismatch	Position	PCR Primer
s1	TATGGAATGAGCATGGGCCA TGG	3	chr1:-130169142	F: ATCTCCGAAGCCAACAGAAG<br>R: TCTGGCATGGTAAGGAGAAAC
	GTTAGAATGTGCATGGGCCG AAG	4	chr12:-93730077	F: GTCAAGTGGCAATGGGTTTC<br>R: CCACAAGATGTCCCTGAGATG
	TCAGGCATGGGCATGGGCCC TGG	3	chr9:-64499502	F: ACACCATTGGCCCAACTAA<br>R: TTAGGAAAGTACAACGGGCTAC
	GTTGGCAGGGGCATGGGCCG CAG	4	chr17:+81565876	F: GGGATACCACCTTGTACCAATAA<br>R: GAAGAGGGCTTGATCCACAA
	TGTGGTACGTGCATGGGCCG TAG	4	chr13:+141566422	F: AACTGTGACAGGGAATCCTAAC<br>R: CCTGTAGCTCACACACAAACTA
	ACTGGAGTGGGCATGGGCAG AGG	3	chr20:+32533869	F: GAGCCAGGATGTGGTTTCTT<br>R: CCTGTATCTGCTGGGTTGTG
s2	ACTTCTGCCCCAAACGGGGC CGG	4	chr19:-39585556	F: CGCAGGAAGCAATGGAAATG<br>R: TGTGTGACAGGGCAAAGAG
	GGGCCTACCCCGAACGGGGC GAG	4	chr7:-43683785	F: CACACACAGTCTCTCTGTTCAC<br>R: TTCCTTCTGCACACCATGTC
	AATGCACCCCCAAACGGGGC AGG	4	chrUN0:-1338853	F: CATGTCTGTCTCTCTGCCTTAT<br>R: TCTCTTCCCTGCTAACTCTTTG
	ATAACCACCCCCAACGGGGC GGG	4	chr8:+14988562	F: CAAGACACAAAGAAGCACCATC<br>R: CCCACCAAGTTATTGGTCTGTA
	TTGTTTACCCCAAAAGGGGC GGG	4	chr1:-18383438	F: AGAGCAGCTTTAGGAACCTTTG<br>R: TCCTGTCTCCCTGCAGTTT
	ATGGCTTGCTCAGACGGGGC CAG	4	chr3:-24783207	F: AGGGAGTGTCCAGGAGTAAA<br>R: TAGCAAGCAGAGAACACTTCAG
